# Comprehensive investigation of the expression profiles of common long noncoding RNAs during microglial activation

**DOI:** 10.5808/gi.22061

**Published:** 2023-03-31

**Authors:** Janghyun Kim, Bora Lee, Young Kim, Byeong C. Kim, Joon-Tae Kim, Hyong-Ho Cho

**Affiliations:** 1 Department of Neurology, Chonnam National University Hospital, Gwangju 61469, Korea; 2 Department of Biochemistry, Chonnam National University Medical School, Gwangju 61469, Korea; 3 Department of Oral Pathology, School of Dentistry, Chonnam National University, Gwangju 61186, Korea; 4 Department of Neurology, Chonnam National University Medical School, Gwangju 61469, Korea; 5 Department of Otolaryngology-Head and Neck Surgery, Chonnam National University Hospital and Chonnam National University Medical School, Gwangju 61469, Korea

**Keywords:** lnc-miRHGs, long noncoding RNA, microglia, M1 microglia activation, M2 microglia activation

## Abstract

Microglia, similar to peripheral macrophages, are the primary immune cells of the central nervous system (CNS). Microglia exist in the resting state in the healthy CNS, but can be activated and polarized into either M1 or M2 subtypes for immune defense and the maintenance of CNS homeostasis by multiple stimuli. Several long noncoding RNAs (lncRNAs) mediate human inflammatory diseases and neuropathologies by regulating their target genes. However, the function of common lncRNAs that contribute to microglial activation remains unclear. Thus, we used bioinformatic approaches to identify common lncRNAs involved in microglial activation *in vitro*. Our study identified several lncRNAs as common regulators of microglial activation. We identified 283 common mRNAs and 53 common lncRNAs during mouse M1 microglial activation processes, whereas 26 common mRNAs and five common lncRNAs were identified during mouse M2 microglial activation processes. A total of 648 common mRNAs and 274 common lncRNAs were identified during the activation of human M1 microglia. In addition, we identified 1,920 common co-expressed pairs in mouse M1 activation processes and 25 common co-expressed pairs in mouse M2 activation processes. Our study provides a comprehensive understanding of common lncRNA expression profiles in microglial activation processes *in vitro*. The list of common lncRNAs identified in this study provides novel evidence and clues regarding the molecular mechanisms underlying microglial activation.

## Introduction

Microglia are the primary immune cells of the central nervous system (CNS). They can be activated and polarized to either M1 (classically activated microglia) or M2 (alternatively activated microglia) subtypes by various pathological stimuli in the CNS, including infection, brain trauma, stroke, and neurodegeneration [1]. Microglia can be stimulated by the bacterial endotoxin lipopolysaccharide (LPS) to an M1 phenotype for the expression of pro‐inflammatory cytokines or by interleukin (IL)‐4 to an M2 phenotype for wound healing and tissue repair [2]. M1 phenotypes are characterized by the production of pro-inflammatory mediators including tumor necrosis factor-α (TNF-α) [3], IL-1β [4], and IL-6 [5], as well as an increased expression of inducible nitric oxide synthase (iNOS), which fuels inflammation. M2 microglial phenotypes release anti-inflammatory cytokines, such as IL-10 [6], as well as transforming growth factor-β (TGF-β) [7], colony-stimulating factor 1, and an enzyme of the urea cycle, arginase-1 (Arg1) [8].

Microglia-mediated neuroinflammation is closely associated with neurodegenerative diseases. It has been shown to contribute to various neurodegenerative diseases, such as Alzheimer’s disease (AD), Parkinson’s disease (PD), multiple sclerosis (MS), and cerebral ischemic injuries. Furthermore, environmental and dietary factors such as chronic stress, circadian rhythms, sleep loss, and the gut microbiota may promote microglia-induced neurodegenerative disorders [9-12].

Some papers have recently reported that microglia during development, aging, and brain injury could be divided into several subtypes, and the transcriptional landscape of microglia subtypes was associated with the local environment [13-15]. Microglia exhibit greater diversity during neuroinflammation and gave rise to several disease-associated phenotypes with distinct gene expression patterns [16].

Long noncoding RNAs (lncRNAs) are transcripts with a minimum length of 200 nucleotides that do not encode proteins. lncRNAs are poorly conserved among different species and exhibit low expression levels [17-19]. Nonetheless, lncRNAs play significant roles in various biological processes, including immunity, inflammation, cell cycle, apoptosis, stem cell pluripotency, and reprogramming, by regulating their target genes [20-24]. lncRNAs can modulate chromatin structure and the function of neighboring and distant genes by interacting with DNA, RNA, and proteins. Some lncRNAs regulate microglial polarization in neurological diseases such as ischemic stroke [25], MS [26], and spinal cord injury [27]. For instance, the lncRNA GAS5 can suppress IRF4 transcription by binding to PRC2 and inhibiting M2 polarization in LPS-stimulated microglia [26]. The lncRNA 1810034E14Rik plays an anti-inflammatory role by regulating p65 phosphorylation [25]. Although the functions of a few lncRNAs during microglial activation have been revealed, the common lncRNAs involved in either M1 or M2 microglial activation remain unknown.

In the present study, we focused on the expression profile of lncRNAs in LPS-induced M1 or IL4-induced M2 microglial activation *in vitro*. We downloaded several sets of RNA expression profiles from the Gene Expression Omnibus (GEO) database and performed a bioinformatic analysis to identify the commonly regulated lncRNAs in microglial M1 or M2 activation in both humans and mice. The list of common lncRNAs identified in this study could be useful to better understand the role of lncRNAs in microglial activation.

## Methods

### Data collection

We searched the PubMed database (http://www.ncbi.nlm.nih.gov/pubmed) for all relevant studies using the keywords “microglia,” “LPS,” “IL-4,” and “activation.” We directly accessed raw gene expression data from LPS-induced M1 activation datasets (GSE75706, GSE79898, GSE80304, GSE90046, GSE105155, and GSE133432 from the GEO database) and IL-4-induced M2 activation datasets (GSE70383 and GSE157891 from the GEO database) via the GEO website (https://www.ncbi.nlm.nih.gov/geo/s) (Table 1).

We downloaded five datasets for mouse M1 microglia and one for human M1 microglia. For GSE75706 and GSE80304, primary microglial cells were treated with LPS (10 ng/mL) for 4 h. For GSE90046, primary microglial cells were treated with LPS (10 ng/mL) for 6 h. For GSE798989, the microglial cell line BV2 was treated with LPS (10 ng/mL) for 4 h. For GSE105155, the microglial cell line BV2 was treated with LPS (1 µg/mL) for 3 h. For GSE133432, induced pluripotent stem cell (iPSC)-derived microglial cells were treated for 24 h with either LPS (100 ng/mL, once) or LPS (2 mg/kg, 3 times).

We downloaded two datasets for mouse M2 microglia. For GSE70383, embryonic stem cell-derived microglia cells were treated with IL-4 (100 U/mL) for 24 h. For GSE157891, primary microglial cells were treated with IL-4 (20 ng/mL) for 48 h.

For validation, we downloaded two datasets: GSE97538 for mouse M1 microglia and GSE158510 for mouse M2 microglia. For GSE97538, primary microglial cells were treated with LPS (5 μg/mL) for 3 h. For GSE158510, primary microglial cells were treated with IL-4 (100 ng/μL) for 24 h.

### Data preprocessing and differential expression analysis

SRR files downloaded from NCBI were converted to FASTQ files using the “fastq-dump” tool in sratoolkit (v22.5.7). FASTQ files were trimmed using Trimmomatic (v0.39) [28] to remove adaptors and low-quality reads. Trimming of reads was conducted in a double pipeline that used two prominent tools for the alignment and quantification of RNA-sequencing (RNA-seq) data: STAR (v2.7.8a) [29] and Subread (v2.0.1) [30].

In the STAR-featureCounts and Subread-featureCounts pipelines, sequencing reads were aligned to the human and mouse genomes (*Homo sapiens*, Genecode v37 and *Mus musculus*, Genecode vM26) using STAR and Subread with the default parameters. Aligned reads were quantified using featureCounts (v1.3.1) [31], as implemented in the R package Rsubread (v1.34.7) according to Genecode transcript annotation (https://www.gencodegenes.org/) [32]. Raw counts were normalized to transcripts per million. Differential expression analysis of lncRNAs and mRNAs between the two conditions was performed using the DESeq2 (version 1.30.1) package [33] in R. We selected the top 10% of lncRNAs and mRNAs from the mouse and human microglial samples using the p-values calculated by DESeq2 (Supplementary Tables 1 and 2). Each RNA-seq dataset was analyzed individually.

### Data visualization

Venn diagrams from RNA-seq data were generated using an online tool provided by VIB and Ghent University (http://bioinformatics.psb.ugent.be/webtools/Venn/). Genomic information on lncRNAs was generated using the Gviz R package (v1.34.1).

### lncRNA-mRNA correlation network

To predict the targets lncRNAs, we conducted *trans*-prediction of lncRNAs with lncRNAs and mRNAs for mouse M1 and M2 microglial activation based on the results of the correlation analysis of lncRNA and mRNA (|correlation| > 0.9 and p < 0.01 for M1 microglia, or |correlation| > 0.85 and p < 0.01 for M2 microglia). The lncRNA-mRNA networks were visualized using Cytoscape 3.8.2. Diamond shapes represent lncRNAs and circular shapes represent mRNAs. Red nodes represent upregulated lncRNAs or mRNAs, and blue nodes represent downregulated lncRNAs or mRNAs.

### Availability of data and materials

All data analyzed in this study were downloaded from the public database in Gene Expression Omnibus (GEO): GSE75706, GSE79898, GSE80304, GSE90046, GSE105155, GSE133432 for M1 microglia and GSE70383, GSE157891 for M2 microglia.

## Results

### Expression profiles of target markers in the microglial polarization state

To identify lncRNAs involved in the regulation of microglial polarization, we searched public GEO datasets according to the following criteria: (1) original studies on microglial activation and (2) expression profiling using high-throughput sequencing. We obtained RNA-seq data for seven LPS-stimulated M1 microglia samples and two IL-4–stimulated M2 microglia samples (Table 1). The LPS-stimulated M1 microglia samples consisted of five mouse and two human samples, and the IL-4–stimulated M2 microglia samples were from mice.

**Fig. 1.**
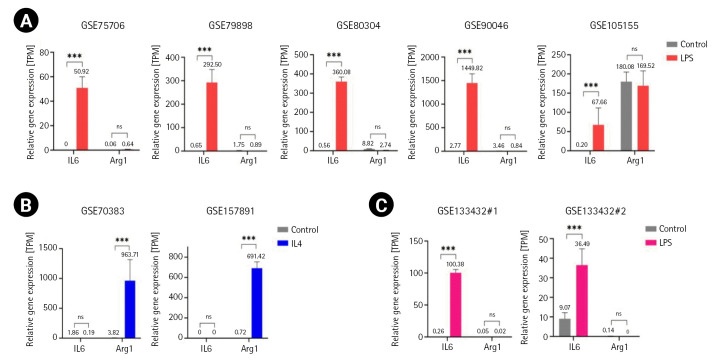
Expression of interleukin 6 (IL-6) and arginase-1 (Arg1) in lipopolysaccharide (LPS)-stimulated (A, C) and IL-4–stimulated microglia (B). Transcripts per million (TPM) were used to measure gene expression levels. Error bars represent standard deviation. The p-value was calculated using the Subread-DESeq2 pipeline. Statistical significance is indicated by asterisks (^***^p < 0.001; ns, not significant).

Next, we confirmed that the RNA-seq data were reliable sources for our aims. We analyzed the expression profiles of protein-coding RNAs in the stimulated and unstimulated samples (Supplementary Table 1). We identified a total of 283 mRNAs (264 upregulated and 19 downregulated) from mouse M1, 915 mRNAs (689 upregulated and 226 downregulated) from human M1, and 26 mRNAs (24 upregulated and 2 downregulated) from mouse M2 microglia RNA-seq datasets (Supplementary Fig. 1). We examined the transcriptome of these RNA-seq samples using IL-6 as the M1 microglial marker gene. IL-6 expression is increased in microglia treated with LPS or amyloid precursor protein [5]. Arg1 was selected as the M2 microglial marker. Arg1 inhibits iNOS activity by competing with the common substrate L-arginine [34]. IL-6 levels were elevated in M1 microglia, whereas M2 microglia exhibited increased levels of Arg1 (Fig. 1A and 1B). As expected, human M1 microglia exhibited increased levels of IL-6, but no change in Arg1 levels as a negative control (Fig. 1C).

Zheng et al. [13] recently reported that microglia can be classified into several clusters, and cytokine and chemokine genes are highly expressed in inflammatory or preactivated microglia (e.g., Il1b, Ccl1, and Ccl4) [13,15,35]. These microglia are similar to M1 microglia in the traditional classification. We checked the expression levels of Il1b, Ccl1, and Ccl4 and found that all these genes showed increased expression in LPS-treated microglia (Supplementary Fig. 2). The results showed that the RNA-seq data were reliable.

Next, we performed differential expression analysis of lncRNAs using these datasets to identify common lncRNAs involved in microglial activation (Supplementary Table 2). Genomic information of the identified lncRNAs was visualized using the Gviz R package.

### Identification of common lncRNAs in the mouse M1 polarization state

**Fig. 2.**
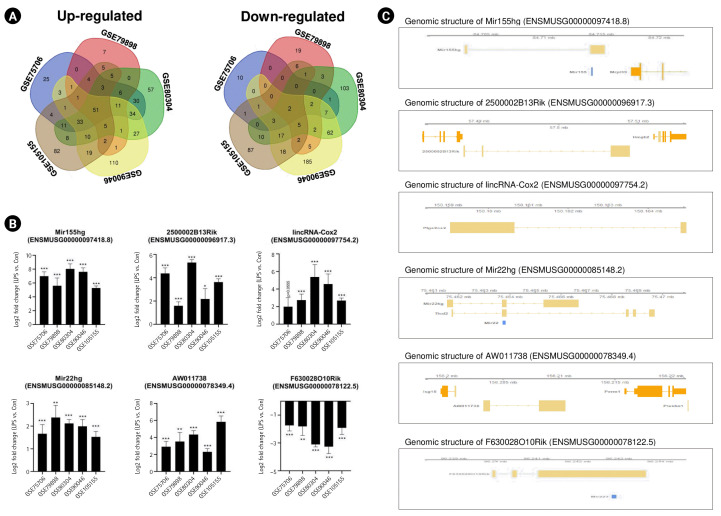
Identification of common long noncoding RNAs (lncRNAs) in the lipopolysaccharide (LPS)-induced mouse M1 polarization state. (A) Venn diagrams displaying the overlapping lncRNAs associated with LPS-stimulated mouse M1 microglia from analyses of the GSE75706, GSE79898, GSE80304, GSE90046, and GSE105155 datasets. (B) Bar graphs showing significant differences in the expression levels of lncRNAs, expressed as log_2_-fold change values, across five different datasets. (C) The identified lncRNAs in their genomic context. Genomic information was visualized with the Gviz package. Mir155hg contains the Mir155 sequence, and Mir22hg contains the Mir22 sequence. F630028O10Rik contains the Mir223 sequence. The p-values were calculated using the Subread-DESeq2 pipeline. Statistical significance is indicated by asterisks (*p < 0.05, ^**^p < 0.01, ^***^p < 0.001).

To determine the common lncRNAs involved in mouse M1 microglial activation, we combined and overlapped the data from the five datasets related to LPS-stimulated microglia. In total, 53 lncRNAs were differentially expressed in mouse samples with and without LPS treatment, with |log_2_FC| > 1.5 and a p < 0.05, from both the STAR-featureCounts and Subread-featureCounts results (Fig. 2A). Among these genes, Mir155hg, 2500002B13Rik, and 6530402F18Rik were upregulated in mouse microglia with M1 activation (Fig. 2B). One of these lncRNAs, Mir155hg, contains the Mir155 sequence (Fig. 2C). Mir155 plays an important role in the induction of an inflammatory microglial phenotype by downregulating Socs1 expression and promoting cytokine production [36,37]. The lncRNA 2500002B13Rik (Nostrill) regulates iNOS gene transcription by interacting with nuclear factor κB (NF-κB) p65 in LPS-stimulated M1 microglia [38]. The lncRNA 6530402F18Rik regulates Pou2fe and Socs1 expression by binding to miR-762 and miR-7648-3p in LPS-treated microglia [39]. As shown in Fig. 2B, Mir22hg and AW011738 showed increased expression in LPS-stimulated microglia, whereas the expression of F630028O10Rik and BC106179 decreased. It has been reported that Mir22hg and AW011738 are involved in immune response and immune system process [25], but the biological functions of these lncRNAs in microglial activation remain unknown.

### Identification of common lncRNAs in the human M1 polarization state

**Fig. 3.**
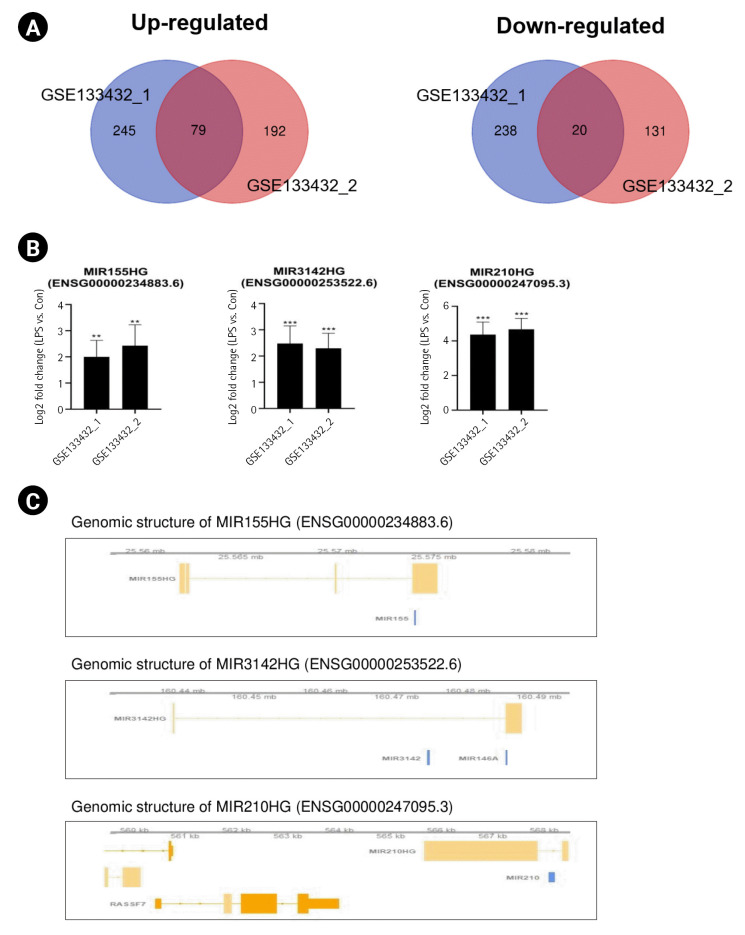
Identification of common long noncoding RNAs (lncRNAs) in the lipopolysaccharide (LPS)-induced human M1 polarization state. (A) Venn diagrams displaying the overlapping lncRNAs associated with LPS-stimulated human M1 microglia from the GSE133432 #1 and GSE133432 #2 analyses. (B) Bar graphs showing significant differences in the expression levels of lncRNAs, expressed as log_2_-fold change values, across 2 different datasets. (C) The genomic context of the identified lncRNAs. Genomic information was visualized with the Gviz package. MIR155HG contains the MIR155 sequence, and MIR3142HG contains the MIR3142 and MIR146a sequences. MIR201HG contains the MIR210 sequence. The p-values were calculated using the Subread-DESeq2 pipeline. Statistical significance is indicated by asterisks (^**^p < 0.01, ^***^p < 0.001).

To identify common lncRNAs involved in human M1 microglial activation, we combined and overlapped data from two LPS-stimulated human microglial datasets. In total, 247 lncRNAs were differentially expressed in human samples with and without LPS treatment, with |log_2_FC| > 1.5 and p < 0.05, from both the STAR-featureCounts and Subread-featureCounts results (Fig. 3A). Among these genes, MIR155HG, MIR210HG, and MIR3142HG were upregulated in LPS-stimulated human iPSC-derived M1 microglia (Fig. 3B). One of the lncRNAs, MIR155HG, was also identified in LPS-stimulated M1 mouse microglia. MIR-210 regulates LPS-stimulated M1 microglial activation by activating NF-κB signaling by targeting SIRT1 and blocking p65 deacetylation [40]. The lncRNA MIR3142HG is a cluster host gene for MIR-3142 and MIR-146a (Fig. 3C). MIR3142HG regulates CCL2 and IL-8 mRNA and protein release during the IL-1β-induced inflammatory response [41].

### Identification of common lncRNAs in the mouse M2 polarization state

**Fig. 4.**
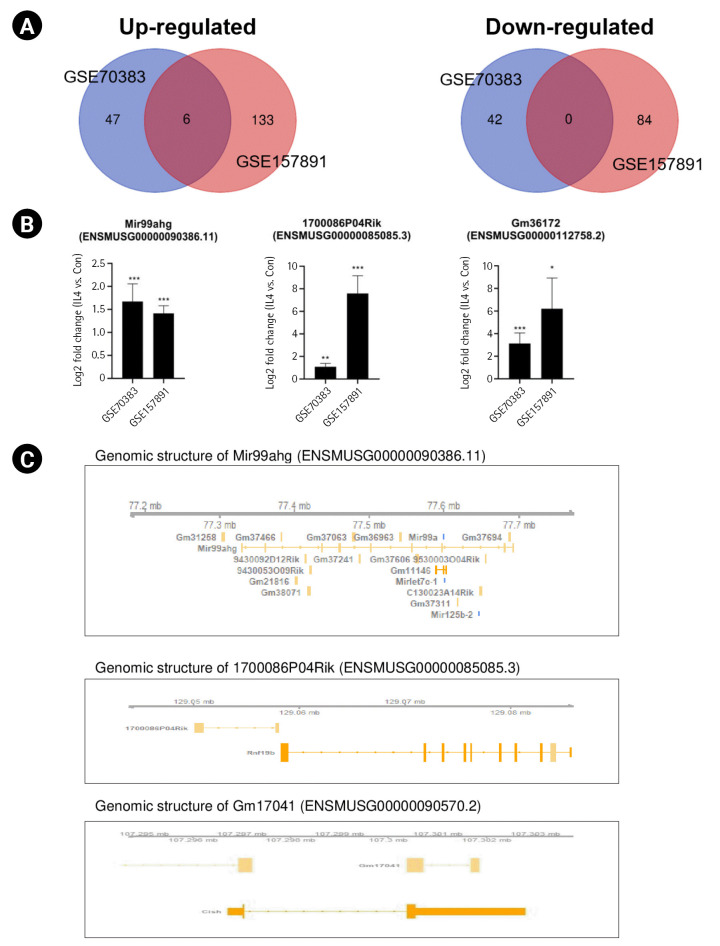
Identification of common long noncoding RNAs (lncRNAs) in the interleukin 4 (IL-4)–induced mouse M2 polarization state. (A) Venn diagrams displaying the overlapping lncRNAs associated with IL-4–stimulated mouse M2 microglia from the analysis of GSE70383 and GSE157891. (B) Bar graphs showing the significant differences in the expression levels of lncRNAs, expressed as log_2_-fold change values, across two different datasets. (C) The identified lncRNAs in their genomic context. Genomic information was visualized with the Gviz package. The lncRNA Mir99ahg contains the Mir99a, Mirlet7c, and Mir125b sequences. The lncRNA 1700086P04Rik and lncRNA Gm36172 have overlapping promoters of Rnf19b and Arg1. The p-value was calculated using the Subread-DESeq2 pipeline. Significance is indicated by asterisks (^*^p < 0.05, ^**^p < 0.01, ^***^p < 0.001).

To identify common lncRNAs involved in mouse M2 microglial activation, we combined and overlapped data from two datasets with IL-4–stimulated microglia. In total, 5 lncRNAs were differentially expressed in mouse samples with and without IL-4 treatment, with |log_2_FC| > 1.5 and p < 0.05, from both the STAR-featureCounts and Subread-featureCounts results (Fig. 4A). Levels of the lncRNA Mir99ahg and 1700086P04Rik were increased in IL-4–stimulated mouse M2 microglial activation (Fig. 4B). Among these lncRNAs, Mir99ahg is a miR-99a, let-7c, and miR-125b-2 cluster host gene (Fig. 4C). It has been reported that miR-99a is specifically expressed in microglia, but not extensively in other immune cell types, and inhibits caspase-3 expression in TGF-β–dependent microglial differentiation [42]. let-7c inhibited microglia-mediated neuroinflammation both *in vivo* and *in vitro* via the translational repression of caspase-3 in ischemic models [43]. The lncRNA 1700086P04Rik is located in the proximal promoter region of ring finger protein 19B (Rnf19b), which is a ring-type E3 ubiquitin-protein ligase (Fig. 4C). It is a hub gene that plays an important role in driving the microglial response to IL-4; however, it has not been previously found to be involved in the immune system and needs to be investigated further [44]. Other lncRNAs—namely, Gm26738, Gm36043. and Gm17041—showed increased levels under conditions of IL-4–stimulated mouse M2 microglial activation. However, the relationship between these lncRNAs and M2 microglial activation remains unclear.

### Validation of the common lncRNAs during microglial activation with independent datasets

We identified a total of 53 common lncRNAs and five common lncRNAs from M1 and M2 microglial activation respectively. To further validate our results, we used independent datasets: GSE97538 for M1 microglial activation and GSE158510 for M2 microglial activation (Supplementary Table 3). We observed similar results in the dependent datasets. In the GSE97538 dataset, MiR155hg, lincRNA-Cox2, and Mir22hg were upregulated in LPS-treated M1 microglia, and BC106179 was downregulated (Supplementary Fig. 3A). In the GSE158510 dataset, Gm36043 and Gm17041 were upregulated in IL-4-treated M2 microglia (Supplementary Fig. 3B).

### Prediction of lncRNA target genes during microglial activation

**Fig. 5.**
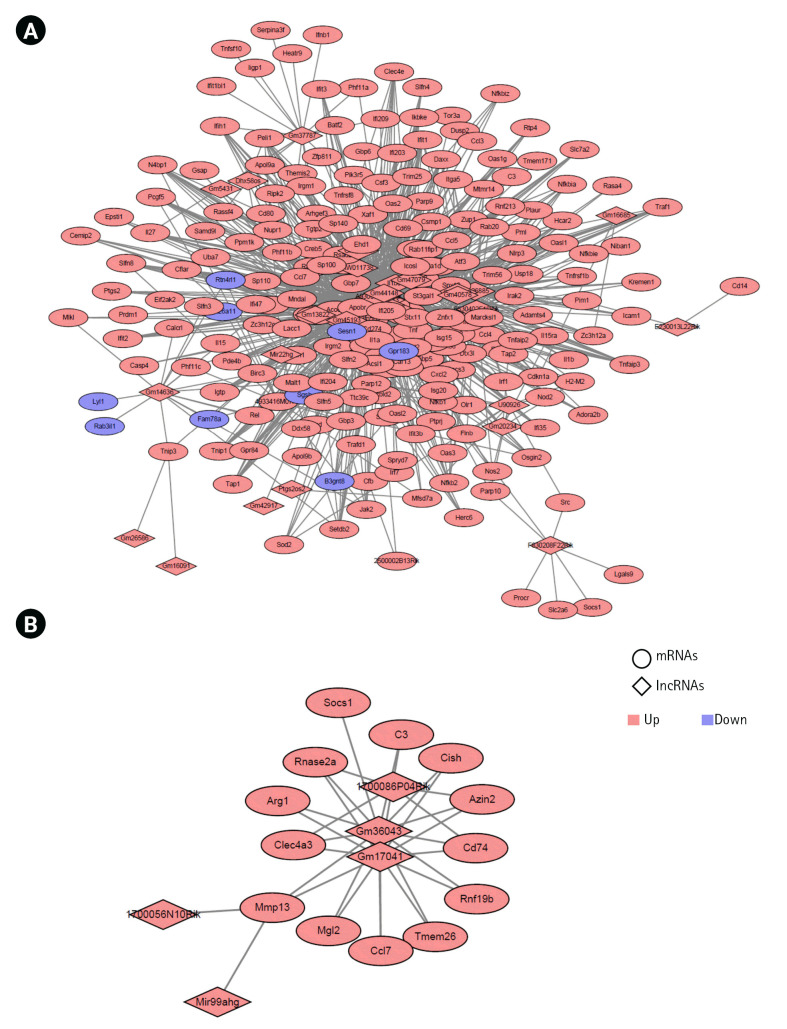
Construction of networks of long noncoding RNA (lncRNAs) and mRNAs in the M1 (A) and M2 (B) microglial activation states. The diamond and circular shapes represent lncRNAs and mRNAs, respectively. Red nodes represent upregulated lncRNAs or mRNAs, and blue nodes represent downregulated lncRNAs or mRNAs.

To reveal the association between the lncRNAs and target mRNAs during microglial activation, we examined the potential *trans*-regulated target genes of lncRNAs [45,46]. We used the Pearson correlation coefficient (PCC) to determine the expression of the 53 common lncRNAs and 283 common mRNAs during mouse M1 activation processes. Among these, the lncRNAs and mRNAs with cutoff values of |correlation| > 0.9 and p < 0.01 were selected. We identified 1,065 co-expressed pairs involving 23 lncRNAs and 211 mRNAs during mouse M1 activation in mice. These co-expressed pairs were used to build the co-expression network using the Cytoscape program (Fig. 5A, Supplementary Table 4). Next, we calculated the PCC for the expression of five lncRNAs and 26 mRNAs in mouse M2 activation processes (GSE70383). We identified six co-expressed pairs with the cutoff value of |correlation| > 0.85 and p < 0.01 (Fig. 5B).

In the co-expression network, A930037H05Rik for M1 activation and Mir99ahg for M2 activation had the highest number of interactions.

## Discussion

Microglia are macrophage-like cells in the CNS that can be activated and polarized to either M1 or M2 subtypes for immune defense and the maintenance of CNS homeostasis. Chronically activated microglia can express immune mediators such as IL-1β, TNF-α, reactive oxygen species, and NO [47]. These immune responses are strictly regulated. However, uncontrolled microglia-mediated neuroinflammation is potentially harmful and one of the features of neurodegenerative diseases, such as AD, PD, MS, and cerebral ischemic injuries [48,49]. Furthermore, microglial activation can be affected by stress-related microbiota, anesthesia-evoked neural responses, and alcohol consumption [12]. lncRNAs are regulators associated with inflammation and immunity. In microglia, a number of lncRNAs have been reported to play crucial roles in microglial activation. For instance, GAS5 suppresses microglial M2 polarization by inhibiting *TRF4* transcription [26], while TUG1 regulates microglial activation by activating *TRIL* expression [50]. However, the common lncRNAs that contribute to microglial activation remain unclear. In the present study, microglial activation-associated lncRNAs were identified by analyzing multiple RNA-seq datasets.

Some lncRNAs are referred to as miRNA-host gene lncRNAs (lnc-miRHGs) because of the presence of pre-miRNAs within their exons or introns [51]. In this study, we identified several lnc-miRHGs, including MIR155HG, MIR210HG, Mir22hg, F630028010Rik, and MIR3142HG, as lncRNAs commonly altered in microglial activation. Among these lnc-miRHGs, MIR155HG was significantly upregulated in both mouse and human M1 microglia (Figs. 2B and 3B). MiR-155 plays an important role in numerous biological processes, including hematopoiesis, inflammation, and immunity, to regulate the expression of target genes [52]. Other lnc-miRHGs, including Mir22hg, F630028010Rik, and MIR3142HG, except MIR210HG, have not previously been reported to be associated with M1 microglial activation. We found that these lnc-miRHGs were regulated by LPS stimulation (Figs. 2B and 3B). We also identified that Mir199ahg, lncRNA 1700086P04Rik, and lncRNA Gm36172 levels increased during IL-4–stimulated microglial activation. Except for Mir199ahg, which is a miR-99a, let-7c, and miR-125b-2 cluster host gene, the role of these lncRNAs in M2 microglial activation is unknown. Thus, their functions during microglial activation require further study.

Interestingly, LPS-induced upregulation of MIR155HG, lincRNA-Cox2, 2500002B13Rik, and MIR210HG appeared to be dependent on NF-κB signaling. MIR155HG is regulated by the pro-inflammatory transcription factor NF-κB and promotes NF-κB activation by decreasing SOCS1 protein levels [36,53]. Moreover, lncRNA-Cox2 and lncRNA 2500002B13Rik are regulated by the NF-κB pathway [38,54]. MIR210HG regulates NF-κB p65 activation by inhibiting Sirt1 activity [40].

Although we identified common lncRNAs during microglial activation, some lncRNAs had unknown biological functions in microglial activation. Thus, the identification of lncRNA target genes is essential for understanding how lncRNAs regulate microglial activation. We identified *trans*-target genes with common lncRNAs and mRNAs during mouse M1 and M2 activation in mice; specifically, we identified a total of 1920 and 25 *trans*-regulated target genes for M1 and M2 microglial activation, respectively (Fig. 5).

In conclusion, we performed a comprehensive analysis of lncRNA expression profiles during microglial activation using public datasets. We identified 288 common lncRNAs during microglial activation *in vitro*. However, our study had some limitations. We used public datasets generated from different samples (primary, embryonic stem cell-derived and iPSC-derived microglia cells, and the BV2 cell line) that were subjected to different stimuli doses and treatment times. Despite these limitations, our study presents crucial findings that enable a more comprehensive understanding of microglial activation and function *in vitro*.

## Figures and Tables

**Table 1.** Characteristics of the datasets selected for the study

GEO No.	Species	Cell type	Treatments	Dataset	
Control	Treat
GSE75706	*Mus musculus*	Primary microglia cells	LPS (10 ng/mL) for 4 h	GSM1964541	GSM1964543
				GSM1964542	GSM1964544
GSE79898	*Mus musculus*	Microglial cell line BV2	LPS (10 ng/mL) for 4 h	GSM2108021	GSM2108024
				GSM2108022	GSM2108025
				GSM2108023	GSM2108026
GSE80304	*Mus musculus*	Primary microglia cells	LPS (10 ng/mL) for 4 h	GSM2124023	GSM2124029
				GSM2124024	GSM2124030
				GSM2124025	GSM2124031
GSE90046	*Mus musculus*	Primary microglia cells	LPS (100 ng/mL) for 6 h	GSM2396057	GSM2396064
				GSM2396059	GSM2396066
				GSM2396062	GSM2396069
GSE105155	*Mus musculus*	Microglial cell line BV2	LPS (1 µg/mL) for 3 h	GSM2823400	GSM2823403
				GSM2823401	GSM2823404
				GSM2823402	GSM2823405
GSE70383	*Mus musculus*	Embryonic stem cell derived microglia (ESdM)	IL-4 (100 U/mL) for 24 h	GSM1726322	GSM1726331
				GSM1726323	GSM1726332
				GSM1726324	GSM1726333
				GSM1726325	
				GSM1726326	
				GSM1726327	
GSE157891	*Mus musculus*	Primary microglia	IL-4 (20 ng/mL) for 48 h	GSM4779218	GSM4779227
				GSM4779228	GSM4779229
GSE133432	*Homo sapiens*	iPSC-derived microglia cells	LPS (100 ng/mL) for 24 h	GSM3908536	GSM3908539
				GSM3908537	GSM3908540
				GSM3908538	GSM3908541
		iPSC-derived microglia cells	LPS (2 mg/kg) for 24 h (3 times)	GSM3908542	GSM3908545
				GSM3908543	GSM3908546
				GSM3908544	GSM3908547
					GSM3908548
					GSM3908549

GEO, Gene Expression Omnibus; LPS, lipopolysaccharide; IL-4, interleukin 4; iPSC, induced pluripotent stem cell.

## Supplementary Materials

Supplementary Table 1. List of differentially expressed mRNAs in each dataset. The log2-fold change values and p-values between the unstimulated and stimulated samples were obtained using the Subread-DESeq2 or STAR-DESeq2 pipeline

Supplementary Table 2. List of differentially expressed long noncoding RNAs (lncRNAs) in each dataset. The log2-fold change values and p-values between the unstimulated and stimulated samples were obtained using the Subread-DESeq2 or STAR-DESeq2 pipeline.

Supplementary Table 3. List of differentially expressed long noncoding RNAs (lncRNAs) in the validation dataset. The log2-fold change values and p-values between the unstimulated and stimulated samples were obtained using the Subread-DESeq2 or STAR-DESeq2 pipeline

Supplementary Table 4. List of long noncoding RNAs (lncRNAs) whose expression correlated with mRNAs in M1 and M2 microglial activation states

Supplementary Fig. 1. Differentially expressed genes during microglial activation and expression profiles of target markers during mouse M1 microglial activation. A Venn diagram showing the number of significantly differentially expressed mRNA profiles during the microglial activation from human and mouse microglia RNA-sequencing data: (A) for mouse M1 microglial activation, (B) for human M1 microglial activation, and (C) for mouse M2 microglial activation.

Supplementary Fig. 2. Expression profiles of target markers during mouse M1 microglial activation. Transcripts per million (TPM) were used to quantify IL-1b, Ccl2, and Ccl4 gene expression levels. Error bars represent standard deviation. The p-value was calculated using the Subread-DESeq2 pipeline. LPS, lipopolysaccharide.

Supplementary Fig. 3. Expression profiles of lncRNAs during mouse M1 and M2 microglial activation. The log2-fold change values and p-values between the unstimulated and stimulated samples were obtained using the Subread-DESeq2 pipeline. Error bars represent standard deviations. LPS, lipopolysaccharide.
