# Supplementary material for: Comprehensive investigation of the expression profiles of common long noncoding RNAs during microglial activation
Source: Genomics Inform. 2023 Mar 31;21(1):e2. doi: 10.5808/gi.22061 (PMC10085744; doi:10.5808/gi.22061)
Supplement: Supplementary Fig. 1. — Differentially expressed genes during microglial activation and expression profiles of target markers during mouse M1 microglial activation. A Venn diagram showing the number of significantly differentially expressed mRNA profiles during the microglial activation from human and mouse microglia RNA-sequencing data: (A) for mouse M1 microglial activation, (B) for human M1 microglial activation, and (C) for mouse M2 microglial activation. [file gi-22061-Supplementary-Figure-1.pdf]

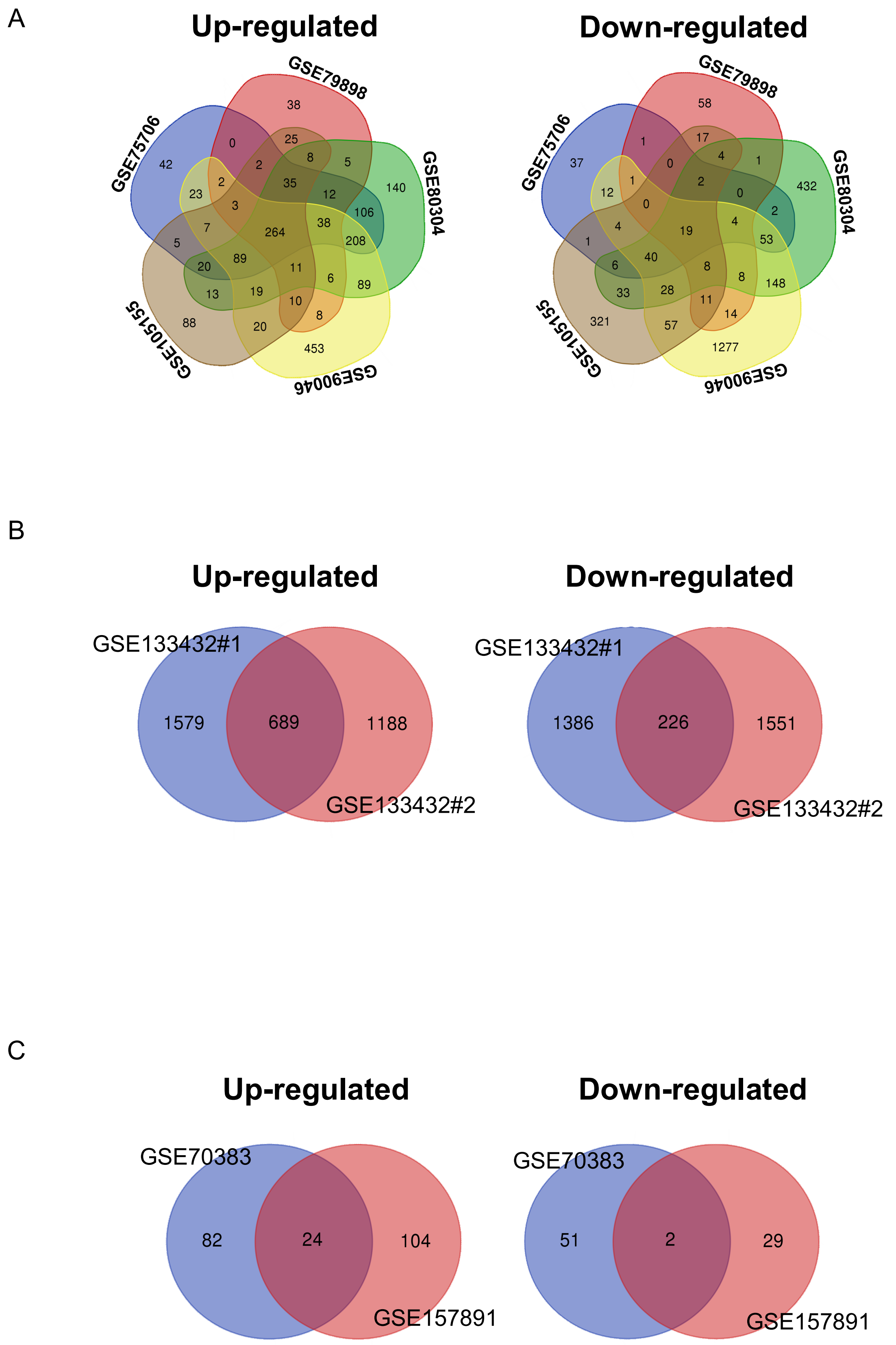

Supplementary Fig. 1. Differentially expressed genes during microglial activation and expression profiles of target markers during mouse M1 microglial activation. A Venn diagram showing the number of significantly differentially expressed mRNA profiles during the microglial activation from human and mouse microglia RNA-sequencing data: (A) for mouse M1 microglial activation, (B) for human M1 microglial activation, and (C) for mouse M2 microglial activation.
