# Supplementary material for: Comprehensive investigation of the expression profiles of common long noncoding RNAs during microglial activation
Source: Genomics Inform. 2023 Mar 31;21(1):e2. doi: 10.5808/gi.22061 (PMC10085744; doi:10.5808/gi.22061)
Supplement: Supplementary Fig. 2. — Expression profiles of target markers during mouse M1 microglial activation. Transcripts per million (TPM) were used to quantify IL-1b, Ccl2, and Ccl4 gene expression levels. Error bars represent standard deviation. The p-value was calculated using the Subread-DESeq2 pipeline. LPS, lipopolysaccharide. [file gi-22061-Supplementary-Figure-2.pdf]

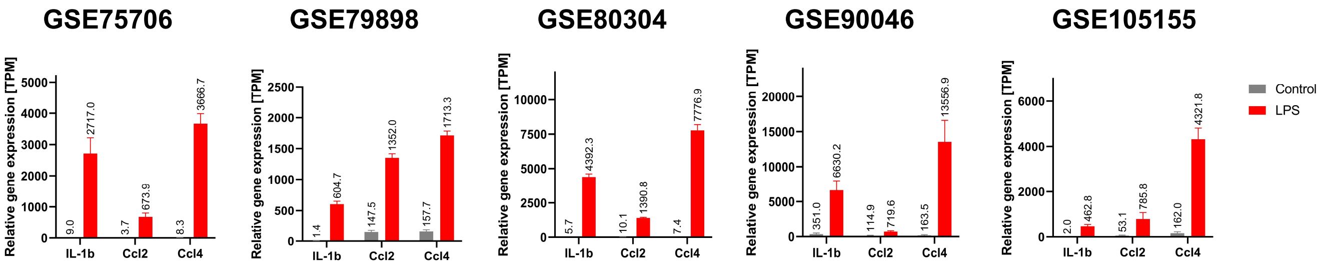

Supplementary Fig. 2. Expression profiles of target markers during mouse M1 microglial activation. Transcripts per million (TPM) were used to quantify IL-1b, Ccl2, and Ccl4 gene expression levels. Error bars represent standard deviation. The p-value was calculated using the Subread-DESeq2 pipeline. LPS, lipopolysaccharide.
