# Supplementary material for: Comprehensive investigation of the expression profiles of common long noncoding RNAs during microglial activation
Source: Genomics Inform. 2023 Mar 31;21(1):e2. doi: 10.5808/gi.22061 (PMC10085744; doi:10.5808/gi.22061)
Supplement: Supplementary Fig. 3. — Expression profiles of lncRNAs during mouse M1 and M2 microglial activation. The log2-fold change values and p-values between the unstimulated and stimulated samples were obtained using the Subread-DESeq2 pipeline. Error bars represent standard deviations. LPS, lipopolysaccharide. [file gi-22061-Supplementary-Figure-3.pdf]

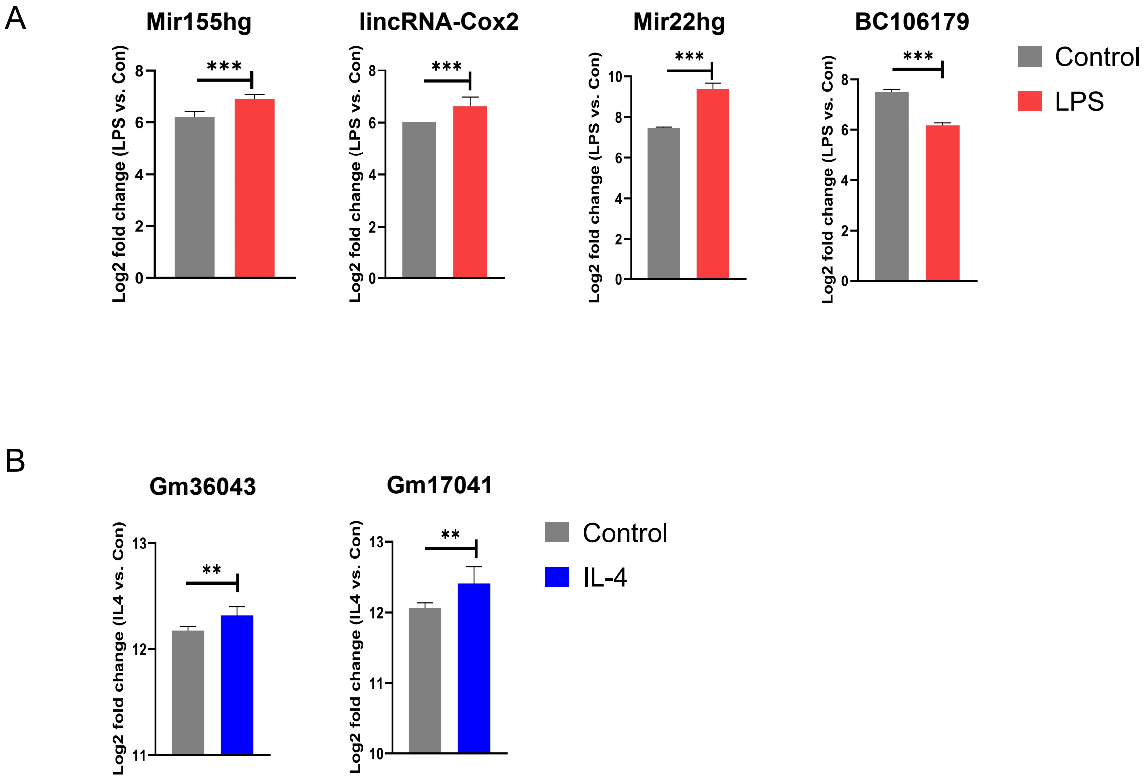

Supplementary Fig. 3. Expression profiles of lncRNAs during mouse M1 and M2 microglial activation. The log2-fold change values and p-values between the unstimulated and stimulated samples were obtained using the Subread-DESeq2 pipeline. Error bars represent standard deviations. LPS, lipopolysaccharide.
